# Pterostilbene Prevents Early Diabetic Retinopathy Alterations in a Rabbit Experimental Model

**DOI:** 10.3390/nu12010082

**Published:** 2019-12-27

**Authors:** Iván Millán, María del Carmen Desco, Isabel Torres-Cuevas, Salvador Pérez, Inés Pulido, Salvador Mena-Mollá, Jorge Mataix, Miguel Asensi, Ángel Luis Ortega

**Affiliations:** 1 Health Research Institute La Fe, Neonatal Research Group, Av. Fernando Abril Martorell 106, 46026 Valencia, Spain; imiya@alumni.uv.es (I.M.); maria.i.torres@uv.es (I.T.-C.); 2 FISABIO Oftalmología Médica, Vitreo-retina unit, Bif. Pío Baroja General Avilés s/n, 46015 Valencia, Spain; Carmen.Desco@uv.es (M.d.C.D.); jorge.mataix@gmail.com (J.M.); 3 Faculty of Pharmacy, Department of Physiology, University of Valencia, Vicente Andrés Estellés Av. s/n, 46100 Burjassot, Spain; Salvador.Perez-Garrido@uv.es (S.P.); ines.pulido@uv.es (I.P.); Salvador.mena@uv.es (S.M.-M.); Miguel.A.Asensi@uv.es (M.A.)

**Keywords:** diabetic retinopathy, polyphenol, pterostilbene, oxidative stress, retinal damage

## Abstract

Oxidative stress generated by diabetes plays a key role in the development of diabetic retinopathy (DR), a common diabetic complication. DR remains asymptomatic until it reaches advanced stages, which complicate its treatment. Although it is known that good metabolic control is essential for preventing DR, knowledge of the disease is incomplete and an effective treatment with no side effects is lacking. Pterostilbene (Pter), a natural stilbene with good antioxidant activity, has proved to beneficially affect different pathologies, including diabetes. Therefore, our study aimed to analyse the protective and/or therapeutic capacity of Pter against oxidant damage by characterising early retinal alterations induced by hyperglycaemia, and its possible mechanism of action in a rabbit model of type 1 diabetes mellitus. Pter reduced lipid and protein oxidative damage, and recovered redox status and the main activities of antioxidant enzymes. Moreover, the redox regulation by Pter was associated with activation of the PI3K/AKT/GSK3β/NRF2 pathway. Our results show that Pter is a powerful protective agent that may delay early DR development.

## 1. Introduction

The World Health Organization estimates that the number of diabetic patients has increased four-fold in the last 40 years [1]. This global increase in diabetes prevalence is associated directly with hyperglycaemia alterations, which are a potent risk factor in macro- and microvascular alterations [2]. Diabetes and poor metabolic control are the leading cause of diabetic retinopathy (DR), an asymptomatic and progressive neuro-vascular complication source of irreversible retinal damage [3].

The retina is one of the most metabolically active tissues of the organism, which make it extremely sensitive to alterations in oxygen levels [4]. Notwithstanding, eye cells are constantly exposed to the effects of reactive oxygen species (ROS) from both the external environment (ultraviolet radiation, metals or cigarette smoke) and endogenous metabolism (alterations in respiration, mitochondrial or viral infections) [5]. This ROS production is counteracted by the existence of enzymes (catalase (CAT), superoxide dismutase (SOD), or glutathione peroxidase (GPx)), molecules with good antioxidant capacity (glutathione, thioredoxin, NADPH) and low-molecular-weight molecules that slow down the action of free radicals (α-tocopherol, ascorbic acid and β-carotene). The imbalance between prooxidants and cellular antioxidants in favour of oxidants causes oxidative stress.

Non-homeostatic increases in glycaemia and ROS overproduction trigger retinal cell demise and phenotypical vascular changes, including retinal ischaemia and permeability, retinal neovascularisation and macular oedema [3,6]. Moreover, different mutations of detoxifying enzymes, such as CAT or SOD, have demonstrated the main role of oxidative stress in DR development [7]. Hence, hyperglycaemia-induced oxidative stress is accepted as one of the main aetiologies to develop diabetes complications like DR [3,8,9].

The treatment indicated for severe or pre-proliferative/proliferative DR stages is photocoagulation with argon laser and immunotherapy, administered via the intravitreal injection of anti-vascular endothelial growth factor (VEGF), associated, or not, with focal laser for diabetic macular oedema. Although newer anti-VEGF drugs have been shown to be cost-effective when used as either monotherapy or combined with focal laser [10], these therapeutic approaches have serious limitations in terms of their use: uncomfortable administration to patients, long-term side effects, high economic cost, and poor therapeutic effectiveness of some administration protocols. Although these therapies delay DR progression and loss of sight, damage to retinal blood vessels and neuronal cell functions is irreversible [3,6]. Therefore, new therapies are needed to prevent or delay DR development.

Polyphenols are a large heterogeneous group of secondary metabolites from plants produced under stress situations. Chemically speaking, these compounds have one aromatic ring or more bearing hydroxyl substituent(s) [11,12]. Polyphenols are the most abundant antioxidants in diet with proven beneficial effects for preventing and treating oxidative damage-related injuries, for example, diabetes, neurodegenerative diseases or cancer [13,14]. Although the antioxidant capacity of Pter is good and it is able to increase cellular and tissue biosynthesis of endogenous antioxidants, the possible benefits in DR remain unclear.

In the present study, we investigated if the in vivo administration of Pter stimulates retinal antioxidant defences and protects against DR development. We also explored the molecular mechanisms involved in retinal damage protection related to the transcription factor nuclear factor erythroid 2 (Nrf2)-mediated antioxidant defence system as a molecular target for Pter through phosphoinositide 3-kinase/protein kinase B (PI3K/Akt) and glycogen synthase kinase 3β (GSK3B) activation.

## 2. Materials and Methods

### 2.1. Animal Model

Thirty-six male New Zealand rabbits (Granja San Bernardo, Navarra, Spain) were randomly divided into three experimental groups (*n* = 12 in each group): non-diabetic (control), diabetic and treated diabetic. Experimental diabetes was induced with alloxan following the protocol described by Alabadí et al. [15]. Briefly, rabbits weighing 2.5–3.0 kg were sedated with intramuscular injection of ketamine (35 mg/kg) (Ketalar ®, Pfizer Inc., Richmond, VA, USA) and xylazine (5 mg/kg) (Dechra Pharmaceuticals PLC, Northwich, UK). Diabetes was induced by injecting alloxan (100 mg/kg) (Sigma-Aldrich, St. Louis, MO, USA) into the marginal ear vein. To prevent hypoglycaemia, glucose 5% (10 mL) was intravenously administered, and drinking water was supplemented with 10% glucose for 24 h. The isotonic solution of Pter phosphate disodium salt (Syncom, Groningen, The Netherlands) was subcutaneously administered to treated diabetic animals daily (74 mg/kg, which equals 50 mg/kg of Pter). Pter treatment started 48 h after inducing diabetes. Animals were maintained on tap water and regular food ad libitum for six weeks.

The work done with animals was approved by the Ethics Committee for Animal Experimentation and Welfare of the University of Valencia (Spain). Housing conditions and experimental procedures were in accordance with European Union (Directive 2010/63/EU) and Spanish (Royal Decree 53/2013) regulations.

### 2.2. Administration and Measurement of Pterostilbene

Blood samples, taken from the central auricular artery of ears, were collected in heparinised tubes after subcutaneous injection of Pter phosphate disodium salt (50 mg/kg of Pter) at different times. Then samples were centrifuged at 1000× *g* for 10 min. Next 150 μL-plasma aliquots were processed by liquid-liquid extraction with ethyl acetate (150 μL) (Sigma-Aldrich, St. Louis, MO, USA). Subsequently, samples were centrifuged at 12,000× *g* for 5 min and supernatants were collected in clean tubes. The liquid-liquid extraction was repeated three times per sample. The supernatant ethyl acetate was evaporated to dryness in a nitrogen stream and the residue was reconstituted in 150 μL of ethanol (Panreac Quimica S.L.U., Castellar del Vallés, Barcelona, Spain Spain). Pter determination was made by UPLC-MS/MS (Waters Acquity UPLC-XevoTQ system) according to Ferrer et al. [16]. Data were acquired and processed using the MassLynx 4.1 and the QuanLynx 4.1 software (Waters Corp., Milford, MA, USA).

### 2.3. Terminal Deoxynucleotidyl Transferase-Mediated dUTP Nick End Labelling (TUNEL) Assay

The quantitative determination of apoptosis in retinas was histologically assessed. For this purpose, animals were euthanized by intravenously administering sodium pentobarbital (100 mg/kg) and perfused with phosphate buffered saline (PBS) (Fisher Scientific, Madrid, Spain). Eyes were enucleated and kept in Davidson’s fixative (8% formaldehyde (Sigma-Aldrich, St. Louis, MO, USA), 30% ethanol, 10% glacial acetic acid (Panreac Quimica S.L.U., Castellar del Vallés, Barcelona, Spain) for 24 h at 4 °C before being transferred to 70% ethanol until use. The paraffin-embedded retinas were sectioned at 5 μm by a microtome (Leica Biosystems, Wetzlar, Germany). Apoptosis was detected using the In Situ Cell Death Detection Kit (Sigma-Aldrich, St. Louis, MO, USA) according to the manufacturer’s instructions, and was then examined by microscopy (Leica DM 4500B, Wetzlar, Germany). Images were analysed by the free NIH ImageJ software (National Institutes of Health, Bethesda, MD, USA).

### 2.4. Biochemistry and Antioxidant Enzymes Activities

The activities of plasma enzymes alanine aminotransferase (ALT) (Abcam, Cambridge, MA, USA), aspartate aminotransferase (GOT) (Abcam, Cambridge, MA, USA), alkaline phosphatase (Abcam, Cambridge, MA, USA), in addition to the plasma levels of total bilirubin (Abcam, Cambridge, MA, USA), albumin (Abcam, Cambridge, MA, USA), chloride (Abcam, Cambridge, MA, USA), blood urea nitrogen (BUN) (Fisher Scientific, Madrid, Spain), creatinine (Abcam, Cambridge, MA, USA), calcium (Abcam, Cambridge, MA, USA), phosphate (Abcam, Cambridge, MA, USA), sodium (Abcam, Cambridge, MA, USA), potassium (Abcam, Cambridge, MA, USA), urea and uric acid (Abcam, Cambridge, MA, USA), were determined by commercial assay kits.

The determinations of CAT, SOD and GPx in the rabbit retinas were made using the Catalase Assay Kit, the Superoxide Dismutase Assay Kit and the Glutathione Peroxidase Assay Kit (Cayman Chemical, Ann Arbor, MI, USA) by spectrophotometry following the manufacturer protocols.

### 2.5. Oxidative Damage

Retinal samples were homogenised in PBS at 30,000 RPM for 30 s (Heidolph Silent Crusher S; Sigma-Aldrich, St. Louis, MO, USA), sonicated for 15 s by keeping pulse duration 5 s ON/5 s OFF (30% amplitude level) and placed in an ice-water bath (Branson SLPe, Branson Ultrasonics Corporation, Danbury, CT, USA). Homogenates were centrifuged at 1500 g for 15 min.

Protein oxidation was evaluated in retinal homogenates by two different methods. Carbonylation was measured by the Rabbit Protein Carbonyl (PC) ELISA Kit (Shanghai BlueGene Biotech CO. Ltd., Shanghai, China) following the manufacturer’s protocol. The 3NO2-Tyr/p-Tyr, m-Tyr/Phe and 3Cl-Tyr/p-Tyr ratios were quantified by UPLC-MS/MS according to Torres-Cuevas et al. [17]. Briefly in retinal homogenates, proteins were precipitated with TCA (10%, *v*/*v*), and pellets were buffered and resuspended in sodium acetate (50 mmol/L, pH 7.2) (Sigma-Aldrich, St. Louis, MO, USA). Next, the protein digestion from tissue extracts was carried out according to Hensley’s method [17]. To finish pronase activity, TCA was used to precipitate it. Then samples were centrifuged (5000 rpm, 4 °C, 5 min) and the supernatant from each sample was injected in the chromatographic system [17].

4-Hydroxy-2-nonenal or 4-hydroxynonenal (4-HNE), a product of lipid peroxidation, was determined by the Rabbit 4-Hydroxynonenal ELISA Kit (Shanghai BlueGene Biotech Co. Ltd., Shanghai, China) following the manufacturer’s protocol.

The reduced/oxidised glutathione (GHS/GSSG), and its precursors γ-l-glutamyl-l-cysteine (γ-Glu-Cys) and S-adenosyl-L-homocysteine (SAH), were analysed by UPLC-MS/MS according to Escobar et al. [18].

The mass spectrometry analysis data were acquired and processed by MassLynx 4.1 and the QuanLynx 4.1 software (Waters Corp., Milford, MA, USA).

### 2.6. Human Retina Endothelial Cells Culture

Human retina endothelial cells (HREC) (Innoprot, Bizkaia, Spain) were maintained in endothelial cell medium (Innoprot, Bizkaia, Spain) with 1% foetal bovine serum (FBS) at 37 °C and 5% CO_2_ in a humidified incubator for optimal nutrition and growth. The used cells were between passages 3 and 5. To perform the glucose cytotoxicity experiments, cells were seeded in fibronectin-coated flasks at a density of 10,000 cells/cm^2^. Cells were supplemented with glucose at normal (5 mM) or high (30 mM) levels, and were treated at different Pter concentrations (DMSO was used as the vehicle at a concentration of 0.1%) 24 h later. Cell growth was analysed using the Countess^®^ Automated Cell Counter (Invitrogen; Thermo Fisher Scientific, Inc., Waltham, MA, USA) after 24 h.

### 2.7. Hydrogen Peroxide Determination

The Amplex Red Hydrogen Peroxide/Peroxidase kit (Fisher Scientific, Madrid, Spain) was used to measure the hydrogen peroxide levels in both the culture media of HREC and the homogenised retina tissue. The procedures were followed according to the manufacturer’s instructions. The fluorescence signal was measured by spectrofluorometry (λexc = 544; λem = 590) (Fluoroskan Ascent, Fisher Scientific, Madrid, Spain).

### 2.8. Gene Expression Analysis. Quantitative Real-Time Polymerase Chain Reaction (qRT-PCR)

To perform the analyses of NAD(P)H dehydrogenase [quinone] 1 (NQO1) expression in HREC, cells were seeded in fibronectin-coated flasks at a density of 10,000 cells/cm^2^ for 24 h. As inhibitors for PI3K kinase, 10 nM of BEZ235 (Sigma-Aldrich, St. Louis, MO, USA), a dual ATP-competitive PI3K and mTOR inhibitor for p110α/γ/δ/β and mTOR(p70S6K) and 20 mM of lithium chloride (Sigma-Aldrich, St. Louis, MO, USA) were used to inhibit GSK3β. These inhibitors were incubated 1 h before Pter treatment. RNA isolation was performed 24 h later.

Total RNA was isolated using the Invitrogen TRIzol Kit following the manufacturer’s instructions. The PrimeScript RT Reagent Kit (Perfect Real Time) (Takara Bio Inc, Shiga, Japan) was employed for cDNA generation following the kit’s indications. Firstly, 500 ng of RNA were used for reverse transcription, done in 2 steps: 15 min at 37 °C and 5 s at 85 °C. Real-time PCR (RT-PCR) was performed by the iQTM5 Multicolor Real-Time PCR Detection System (Bio-Rad, Richmond, CA, USA). The threshold cycle (CT) was determined and the relative gene expression was expressed as follows: fold change = 2 − Δ(ΔCT), where ΔCT = CT target − CT reference transcript, and Δ(ΔCT) = ΔCT treated − ΔCT control. β-ACTIN was used as the reference transcript. The employed specific primers were (5′ to 3′): NQO1, forward CCATGGTCGGCAGAAGAG and reverse, GATGGGATTGAAGTTCATGG; β-ACTIN, forward, GACCCAGATCATGTTTGAGA and reverse, AGGGCATACCCCTCGTAGAT.

### 2.9. NRF2 Immunocytochemistry

To perform the NRF2 translocation analyses, HREC were seeded in fibronectin-coated LabTek II chambers (Nalge Nunc International, Rochester, NY, USA) and treated with Pter 5 uM (DMSO as the vehicle was used at a concentration of 0.1%) 24 h later. Cells were incubated for 0, 5, 6 or 8 h and were washed with PBS and fixed with 4% paraformaldehyde (Sigma-Aldrich, St. Louis, MO, USA) for 30 min. Then cells were incubated with 1% Triton X-100 in PBS for 10 min to permeabilise cell membranes, followed by incubation with 3% bovine serum albumin (BSA) and 5% FBS in PBS for 1 h. Cells were incubated with 1: 250 dilution of primary antibodies against NRF2 (Cell Signaling, Danvers, MA, USA) overnight at 4 °C, followed by the Alexa Fluor^®^ secondary antibody (Fisher Scientific, Madrid, Spain) for 1 h at room temperature in the dark. Nuclei were stained with 4,6-diamidino-2-phenyindole, dihydrochloride (DAPI) (Fisher Scientific, Madrid, Spain) and then examined under a Leica TCS SP2 confocal microscope (Leica, Wetzlar, Germany).

### 2.10. Protein Extraction and Western Blot Analysis

Cells were rinsed twice with cold PBS and proteins were extracted by scraping in ice-cold lysis buffer (Cell Signaling, Danvers, MA, USA) containing a protease and phosphatase inhibitors cocktail (Sigma-Aldrich, St. Louis, MO, USA). Tissue samples were homogenised in ice-cold lysis buffer at a ratio of 100 mg/mL. Equal amounts of the extracted proteins (40 µg/condition) were resolved using Mini-PROTEAN^®^ TGX Stain-Free™ Precast Gels (Bio-rad, Richmond, CA, USA) before being transferred to PVDF membranes by the Trans-Blot^®^ Turbo™ Transfer System (Bio-rad, Richmond, CA, USA). Blots were incubated overnight at 4 °C with primary antibodies p-PI3K (Cell Signaling, Danvers, MA, USA), PI3K (Cell Signaling, Danvers, MA, USA), p-AKT (Cell Signaling, Danvers, MA, USA), AKT (Cell Signaling, Danvers, MA, USA), p-GSK3β (Abcam, Burlingame, CA, USA), GSK3β (Abcam, USA) and NQO1 (Abcam, USA). Blots were incubated for 1 h at room temperature with the appropriate peroxidase-conjugated secondary antibody HRP-linked antibody (Cell Signaling, Danvers, MA, USA). Blots were visualised using a chemiluminescence detection kit ECL Western blotting substrate (Fisher Scientific, Madrid, Spain). Signals were captured by the ChemiDoc™ XRS + Imaging System (Bio-rad, Richmond, CA, USA). The density of bands was measured by version 2.0.1 of the Image Lab Software™ (Bio-rad, Richmond, CA, USA).

### 2.11. Statistical Analysis

Both the calculations and graphical representations were created with the Microsoft Office Excel 2016 programme and GraphPad Prism 5.0. The GraphPad Prism 5.0 for Windows software was used for the statistical analysis of the results herein presented.

A one-way ANOVA was employed to determine the differences among groups, followed by Newman–Keuls or Tukey´s multiple comparison tests, whenever appropriate. The null hypothesis was rejected for all the values in the tests in which the F value was significant with a *p*-value less than 0.05.

## 3. Results

### 3.1. Phosphorylated Pterostilbene Disodium Salt as a Source of Pterostilbene

**Figure 1:**
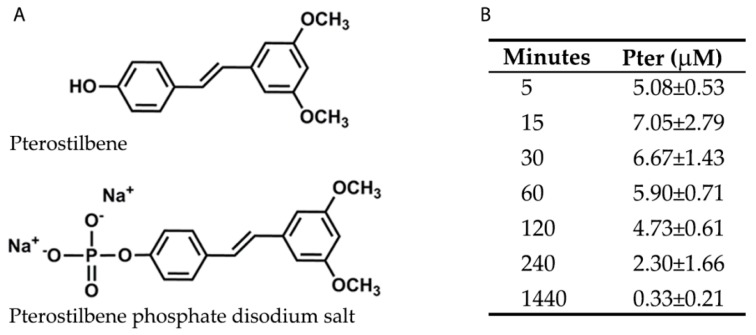
Chemical structures and plasma levels of Pter after administering Pter phosphate disodium salt. The pharmaceutical form of disodium salt of Pter phosphate was used to facilitate its daily subcutaneous administration in vivo. (**A**) Chemical structures of Pter and Pter phosphate disodium salt. (**B**) A subcutaneous injection of Pter phosphate disodium salt (Pter 50 mg/kg) was administered dissolved in water (25 mg/mL). Blood was collected at different times and Pter was quantified in plasma by the UPLC-MS/MS analysis. The results are the means ± S.D. of four different rabbits.

To perform Pter administration and to study its possible benefits against glucose damage in vivo, phosphorylated Pter disodium salt was used (Figure 1A). The phosphate group in position 4′ increased solubility in water and reached active biologically concentrations in plasma during the first 4 h after subcutaneous administration (Figure 1B). Pter was also determined in the plasma from the treated rabbits for six weeks, 24 h after the last injection (0.60 ± 0.19 μM). Non-significant differences were observed in the values obtained after a single administration (0.33 ± 0.21 μM). Our results show that Pter was released from the phosphorylated Pter disodium salt and was detected in plasma at a stable concentration for at least 2 h. Besides, stilbene did not accumulate after six weeks of subcutaneous administrations.

### 3.2. Pterostilbene Reduces the Harmful Retinal Effects of Hyperglycaemia

**Figure 2:**
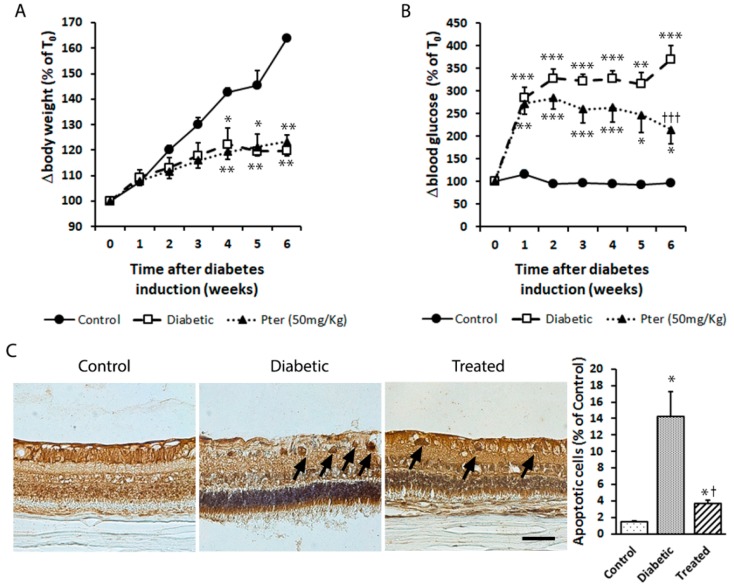
Type 1 diabetes mellitus was induced in rabbits by alloxan injection and rabbits were treated daily with Pter phosphate disodium salt (50 mg/kg of Pter) for six weeks (Treated). (**A**) Weight (BW) and (**B**) blood glucose (BG) levels were evaluated weekly. (**C**) Retinal apoptotic cell death was examined and quantified by TUNEL staining (black bar = 50 µm) six weeks after diabetes induction. Black arrows mark apoptotic cells. Data are presented as mean ± S.E.M. (at least *n =* 5 in each group). An ANOVA, followed by Tukey’s post hoc test, was used to assess significant differences between experimental conditions. * *p* < 0.05; ** *p* < 0.01; *** *p* < 0.001 versus the control group. † *p* < 0.05; ††† *p* < 0.001 versus the diabetic group.

The protective effect induced by Pter was observed in the rabbit retinas in an experimental diabetes type 1 model. The diabetic and Pter-treated diabetic rabbits underwent significant weight loss compared to the control condition (Figure 2A). The differences in body weight started in the second week and increased in both groups throughout the experimental period. Moreover, diabetes induced increased blood glucose and Pter significantly reduced hyperglycaemia, although it was unable to lower it to the control values (Figure 2B). However, polyphenol treatment resulted in a significant drop in ganglion cell death apoptosis shown for the diabetes condition according to TUNEL staining (Figure 2C).

### 3.3. Haematological Parameters and Pterostilbene Toxicity

The biochemical determinations are presented in Table 1. In the diabetic rabbits, ALT and alkaline phosphatase were significantly higher than in the controls and treated animals. Albumin and alkaline phosphatase significantly lowered in the treated animals. No significant differences were observed in the rest of analysed toxicological parameters. Hence, statistical differences were considered to fall within the range of normal variability, regardless of Pter administration and with no toxicological significance.

### 3.4. Pterostilbene Inhibits Considerable Glucose-Oxidative Damage and Reduces Hydrogen Peroxide Production In Vivo

**Figure 3:**
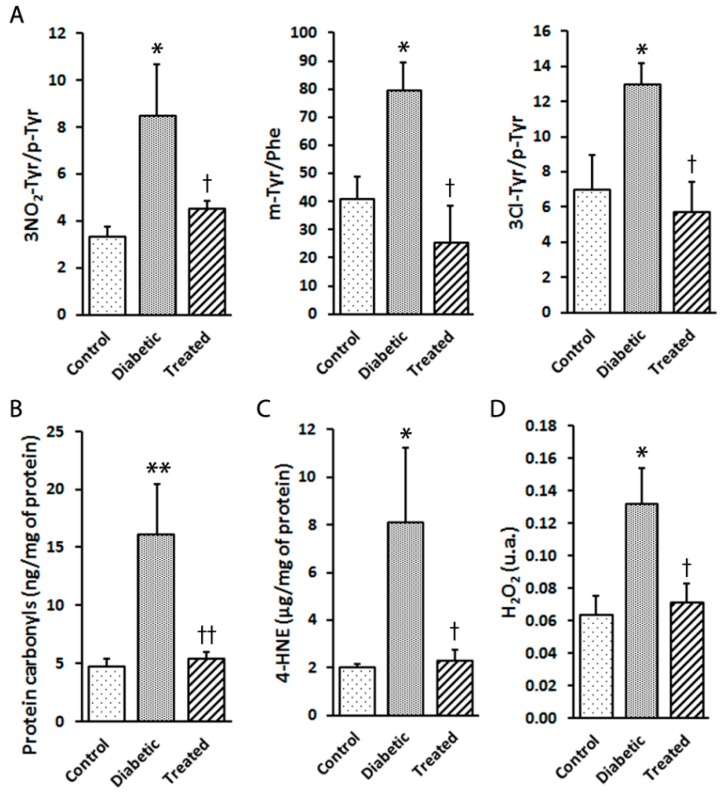
Effect of pterostilbene treatment on the oxidation of proteins and lipids, as well as hydrogen peroxide production, in the rabbit retinas. Retinal samples from the control, diabetic and diabetic and Pter-treated rabbits were obtained six weeks after inducing diabetes. (**A**) The 3NO2-Tyr/p-Tyr, m-Tyr/Phe and 3Cl-Tyr/p-Tyr ratios were quantified by UPLC-MS/MS. (**B**) Carbonylation was measured in retinal homogenates by ELISA. (**C**) Lipid peroxidation was determined by ELISA. (**D**) Hydrogen peroxide was established in retina homogenates by spectrofluorometry. Data are presented as mean ± SEM (at least *n =* 4 in each group). Data were analysed by a one-way ANOVA followed by Newman-Keuls test. * *p* < 0.05; ** *p* < 0.01 versus the control group. † *p* < 0.05; †† *p* < 0.01 versus the diabetic group.

It is well-known that hyperglycaemia is a potent inductor of oxidative stress and is able to trigger increased cell damage, loss of function and DR development in the retina. Here we show effects of diabetes, such as a significant increase in retinal protein and lipid oxidation. As observed in Figure 3, Pter significantly diminished the oxidative damage induced by hyperglycaemia exposure. The effect on protein was evaluated as the mTyr/Phe, 3NO2Tyr/p-Tyr, and 3Cl-Tyr/p-Tyr ratios (Figure 3A) and carbonylation levels (Figure 3B), and lipid damage was assessed by 4-HNE quantification (Figure 3C). Moreover, hydrogen peroxide, a biologically derived stable oxidant intermediate, can inflict damage to ocular tissues by disrupting cellular structure and function. The analyses of the retinas from diabetic animals presented high hydrogen peroxide levels, while Pter lowered them (Figure 3D) to the control values, which shows the high antioxidant capability of Pter in vivo.

### 3.5. Pterostilbene Prevents Retinal Damage by Modulating Antioxidant Defences

**Figure 4:**
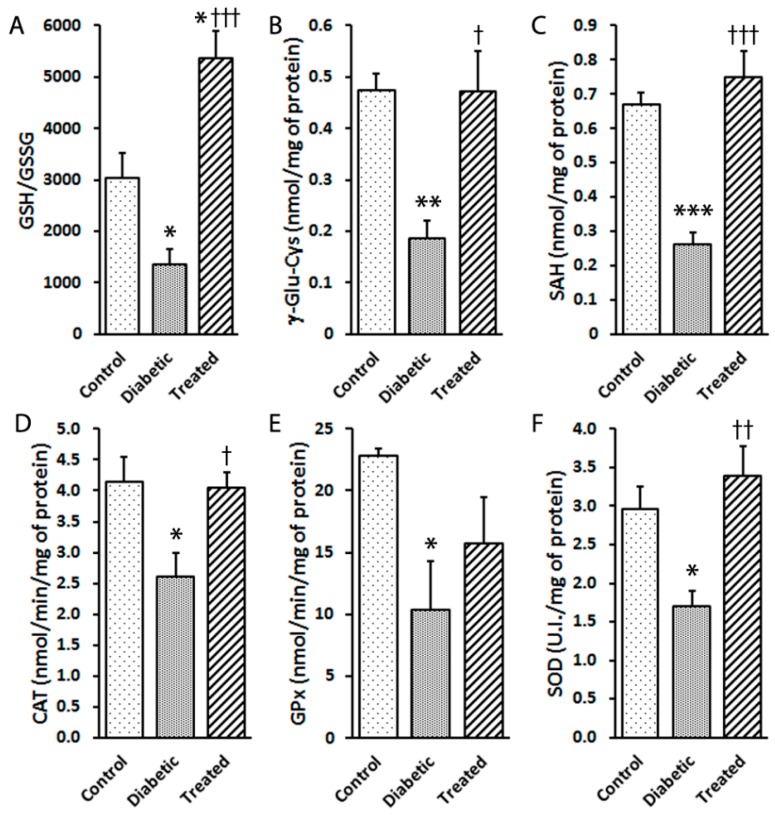
Effect of pterostilbene on antioxidant machinery activation in diabetic rabbits. Type 1 diabetes mellitus and Pter treatment were performed as described in Figure 2. The analyses of retinal (**A**) GSH/GSSG, (**B**) γ-Glu-Cys and (**C**) SHA were performed by UPLC-MS/MS. The enzymatic activities of (**D**) catalase (CAT), (**E**) glutathione peroxidase (GPx) and (**F**) superoxide dismutase (SOD) were determined by spectrophotometry. Data are presented as mean ± SEM (at least *n =* 4 in each group). Statistical analyses were performed by a one-way ANOVA followed by Tukey’s test. * *p* < 0.05; ** *p* < 0.01; *** *p* < 0.001 versus the control group. † *p* < 0.05; †† *p* < 0.01; ††† *p* < 0.001 versus the diabetic group.

The retinas of the diabetic animals showed a depleted glutathione redox status, expressed as the GSH/GSSG ratio, but protected by Pter treatment (Figure 4A). Similar effects were observed after analysing γ-Glu-Cys, a substrate used for GSH biosynthesis (Figure 4B), and S-adenosyl-l-homocysteine (SAH), a component of the transsulphuration pathway (Figure 4C).

To identify the contribution of the enzymatic antioxidant defence mechanism, the enzymatic activities of CAT (Figure 4D), GPx (Figure 4E) and SOD (Figure 4F) were measured. Hyperglycaemia diminished the activities of CAT, GPx and SOD compared to the controls. These effects on enzymatic activities were reverted by Pter treatment (Figure 4D–F). These results concurred with increased vulnerability to oxidant damage and hydrogen peroxide production (Figure 2C and Figure 3).

### 3.6. Molecular Mechanisms Involved in the Antioxidant Role of Pterostilbene

**Figure 5:**
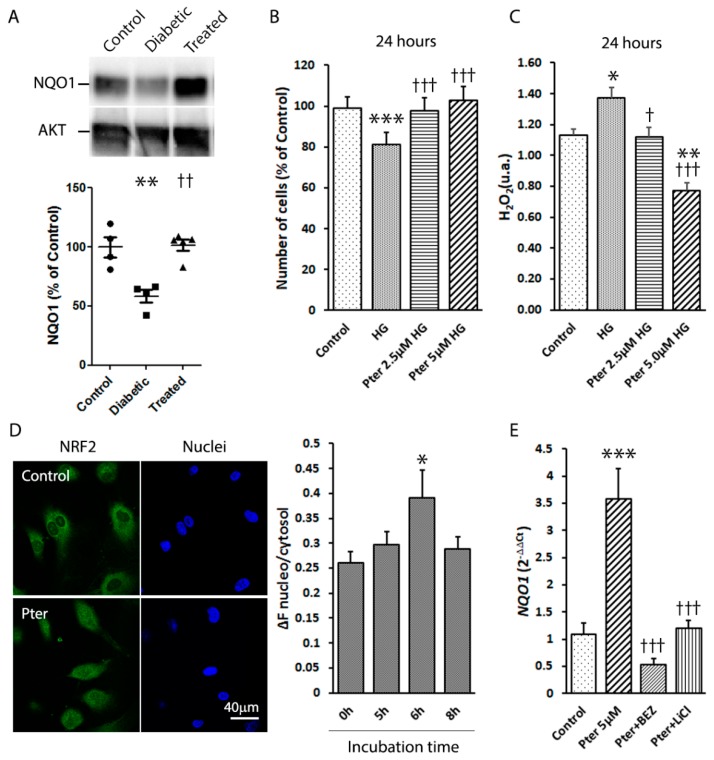
Effect of pterostilbene on NRF2 activation. (**A**) A Western blot was used to detect the expressions of NQO1 in retinas. Data are presented as mean ± SEM (at least *n =* 4 in each group). A one-way ANOVA and a Newman-Keuls multiple comparison test were used. ** *p* < 0.01; versus the control group; †† *p* < 0.01 versus the diabetic group. (**B**) HREC were treated for 24 h with Pter (2.5 μM and 5 μM) at high (30 mM) glucose concentrations. Statistical analyses were performed by a one-way ANOVA, followed by Tukey’s test. *** *p* < 0.001 versus the control group. ††† *p* < 0.001 versus the high glucose group. (**C**) Hydrogen peroxide was determined in HREC medium after 24 h of incubation with Pter (2.5 μM and 5 μM) at high (30mM) glucose concentrations. An ANOVA followed by Tukey’s post hoc test, was used to assess significant differences between the experimental conditions * *p* < 0.05; ** *p* < 0.01; versus the control group. † *p* < 0.05; ††† *p* < 0.001 versus the high glucose group. (**D**) NRF2 translocation was detected by confocal microscopy in HREC. Cells were incubated with Pter 5 μM and fixed with 4% paraformaldehyde at 0, 5, 6 and 8 h. NRF2 was detected by immunocytochemistry and NRF2 translocation was evaluated by the ImageJ software. A representative image after 6 h of incubation with Pter. Data were analysed by a one-way ANOVA, followed by Tukey’s test. * *p* < 0.05 versus the control group (0 h) (**E**) HREC were incubated in the presence of DMSO (control), Pter 5 μM, Pter (5 uM) + BEZ235 (10 nM), and Pter (5 uM) + LiCl (20 mM) for 24 h. The RNA levels of *NQO1* were determined by qRT-PCR normalised to the *β-Actin* mRNA levels. Data are presented as mean ± SEM (at least *n =* 3 in each group). Differences among groups were assessed by a one-way ANOVA, followed by Tukey’s test. *** *p* < 0.001 versus the control group. ††† *p* < 0.001 versus the Pter group.

NRF2 pathway activity is overwhelmed by oxidative stress in diabetes. Thus, in order to study the potential role of Pter on NRF2 activation, the downstream NQO1 expression was analysed on retinas of control, diabetic and Pter-treated diabetic animals. According to the above-observed antioxidant protection, NRF2 activation triggered an increase in NQO1 expression in the presence of Pter (Figure 5A). To evaluate the protective role of Pter against oxidative stress induced by high glucose exposure, HREC were incubated with different concentrations of polyphenol, plus 5 mM (control) or 30 mM glucose (high glucose). The cell number significantly lowered in the presence of 30 mM glucose from the control group. Incubation, with previously determined non-cytotoxic Pter concentrations (2.5 µM and 5 µM), had a protective effect against high glucose cytotoxicity on HREC after 24 h of incubation (Figure 5B). The analyses of the HREC cells exposed to high glucose levels of the culture medium revealed that the release of hydrogen peroxide increased, while Pter lowered the levels of this ROS to the control values (Figure 5C). Furthermore, the nuclear to cytoplasmic NRF2 ratio was used as an index of NRF2 translocation (Figure 5D). NRF2 activation was maximum after 6 h of Pter incubation in HREC, which confirmed the key NRF2-dependent antioxidant role of Pter.

**Figure 6:**
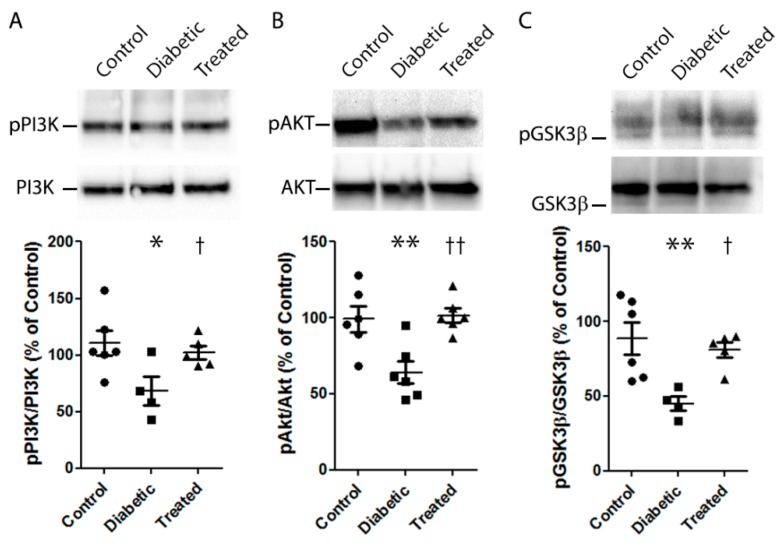
Effect of pterostilbene on the PI3K/AKT/GSK3β/NRF2 pathway. Type 1 diabetes mellitus and Pter treatment were performed as described in Figure 2. A Western blot was used to detect the expressions of pPI3K/PI3K (**A**), pAKT/AKT (**B**), pGSK3β/GSK3β (**C**). Data are presented as mean ± SEM (at least *n =* 4 in each group). An ANOVA, followed by a Newman–Keuls post hoc test, was used to assess any significant differences between the experimental conditions. * *p* < 0.05; ** *p* < 0.01 versus the control group. † *p* < 0.05; †† *p* < 0.01 versus the diabetic group.

The activation of the PI3K/Akt/ GSK3β pathway is deemed an important NRF2 function trigger [19,20,21,22]. Thus, in order to investigate the molecular pathway by which Pter stimulates antioxidant protective mechanisms and reduces retinal damage, the action of PI3K and GSK3β was modulated in HREC. The mRNA levels of *NQO1* were lowered by inhibiting PI3K (BEZ235) and GSK3β (lithium chloride, LiCl), and by reversing Pter-induced NRF2 activation (Figure 5E), which revealed the critical role of this pathway in Pter antioxidant action. To evaluate the effect of this stilbene in vivo, the status of the PI3K/AKT/GSK3β/NRF2 pathway was analysed on the retinas of the experimental animals. In parallel to the recovered antioxidant defences shown in Figure 4 and the increased NQO1 expression noted after Pter treatment (Figure 5A), the phosphorylation levels of PI3K, AKT and GSK3β in the diabetic rabbit retinas lowered (Figure 6), while Pter treatment recovered the values to the control levels.

## 4. Discussion

Nowadays, DR therapies act in advances stages of this disease by showing efficacy problems and causing side effects. Lack of an effective therapy, together with the fact that inflicted retinal damage is irreparable, mean that it is necessary to find new strategies to prevent DR onset. Oxidative stress, which increases with hyperglycaemia, is considered one of the main aetiologies for diabetes complications like DR to develop [8,9]. Free radicals are capable of damaging cellular DNA, proteins and lipids, which leads to altered cellular functions. Many studies have revealed that nutraceuticals capable of neutralising free radicals are effective in reducing the severity of diabetic complications like DR [3]. Thus, for example, a protective effect has been observed for DR and other retinopathies with resveratrol [23,24,25], epigallocatechin gallate [26,27], curcumin [28,29] and quercetin [30,31], among others. Their actions have been attributed to their capacity to modulate different molecular targets, including sirtuins like SIRT1 [32], kinases such as MAPK [33,34,35], transcription factors like Nrf2/ARE [36,37], nuclear factor kappa B (NF-kB) [34] and others that provide antioxidant and anti-inflammatory protection [3]. We have demonstrated the protector effect of Pter as an antioxidant in different experimental models [38]. Sheng and Rong have shown that Pter delays DR progression by alleviating the inflammation and oxidation of biomolecules using an in vitro model of human retinal endothelial cells [39]. However, the effect of Pter on retinas in animal models has not yet been explored.

DR is a complex heterogeneous pathology and, to date, no animal model is able to reproduce all its pathological characteristics. Strain C57BL/6-Ins2^Akita^/J (Jackson Lab) is a monogenic mouse model based on the spontaneous mutation in the insulin 2 gene that leads to the incorrect folding of the insulin protein by producing toxicity in pancreatic β cells. The Akimba (Ins2^Akita^ × VEGF+/−) model shows typical vessel alterations of advanced DR stages. Streptozotocin (STZ) and alloxan, used to induce type 1 diabetes mellitus in rats, mice or rabbits, are glucose analogues that penetrate pancreatic β cells and induce their demise [40]. Unlike other authors who have used STZ to induce diabetes in rats [41,42,43,44], we observed no significant difference in weight (Figure 2A) and blood glucose levels (Figure 2B) between diabetic and Pter-treated diabetic rabbits. This can be understood by the fact that the cytotoxic effect of STZ and alloxan is achieved by different molecular pathways [40], or by an insufficient Pter concentration, which was able to reverse hyperglycaemia with alloxan induction in our experimental model.

Polyphenols are characterised by poor bioavailability, mainly due to low solubility and rapid metabolism, which limit their clinical use. We decided to add a phosphate disodium group in position 4′ to increase Pter solubility (Figure 1A). The hydroxyl group in position 4′ is essential for its activity [45]. For this reason, we determined Pter blood levels after the subcutaneous administration of Pter phosphate disodium salt to confirm phosphatases action and the biological action of Pter (Figure 1B).

Pter was injected daily into different locations on the back to avoid the likelihood of inducing oesophagus damage by an oral probe and skin damage appearing, e.g., granulomas. The non-toxic Pter doses of 50 mg/kg (Table 1) were similar to those employed in other works in which Pter proved biologically effective [44,46,47,48]. Our conclusion about Pter toxicity is consistent with previous studies in which mice were fed with up to 3 g/kg of pterostilbene per day for 28 days (the equivalent doses in a human would be 17.67 g/day) [46]. In addition, Pter is also safe in humans in, for example, a prospective clinical trial, where patients with hypercholesterolaemia were treated with Pter for an average 52-day duration, which demonstrates that it is safe up to 250 mg/day [49]. Moreover, the treatment of healthy volunteers with 450 mg of *Pterocarpus marsupium* extract did not produce any sign of toxicity [50]. According to our results, some incidence of liver function abnormalities was observed in diabetic rabbits, but not in the Pter-treated diabetic rabbits (Table 1). Regarding kidney protection, other authors have shown kidney damage in diabetic animal models [51], but our results did not show this alteration (Table 1). Surely we should observe this complication at longer times.

Plenty of evidence has demonstrated that retinal ganglion cell apoptosis occurs in the neuroretinas of diabetic patients in early stages, regardless of disease duration or the appearance, or not, of phenotypical damage by using spectral-domain optical coherence tomography [52,53]. The present study demonstrates that an effective Pter dose is an efficient protector against retinal apoptosis induced by hyperglycaemia (Figure 1). Moreover, as Pter administration does not influence body weight and blood glucose levels, any beneficial effects should be attributed to a direct effect of Pter on the retina. The demise of retinal neural cells may be induced by hyperglycaemia-generated hydrogen peroxide [54], and Pter reduces its cytotoxicity, oxidative stress and apoptosis in different cellular models like HepG2 cells [55]. According to our results, Pter decreased the hydrogen peroxide production induced by high glucose levels in vivo (Figure 3D) and in vitro (Figure 5C). Furthermore, daily Pter systemic administrations to diabetic rabbits prevented oxidative damage to retinal proteins (Figure 3A,B) and lipids (Figure 3C) induced by hyperglycaemia.

The antioxidant capability of Pter has been previously shown in several experimental models [37,38,55,56,57,58] and our results well agree with the previously reported effect of Pter on NRF2 activation [37,38,57], one of the main redox homeostasis regulators and an appealing therapeutic target for DR [59]. Under physiological conditions, NRF2 is sequestered mostly in the cytosol and degraded through ubiquitination. NRF2 activation triggers its translocation into the nucleus, binds to antioxidant response element (ARE) sequences and increases the expression of phase II detoxifying and antioxidant genes, such as heme oxygenase 1 (HO-1), NQO1, thioredoxin reductase (TrxR), peroxiredoxins (Prxs), SOD, CAT, GPx, GSH reductase (GR), GSH S-transferase (GST) and glutamate-cysteine ligase (GCL) [60,61]. Consequently, in the present work, NRF2 action rapidly eliminated ROS and played a key defensive role in cell homeostasis. Accordingly, Pter treatment improved the GSH/GSSG ratio, and increased not only γ-GC and SAH concentrations (Figure 4A–C), but also the recovery of the main antioxidant enzyme activities CAT, SOD and GPx (Figure 4D–F) in diabetic rabbit retinas. Moreover, NRF2 activation increased the protein levels of NQO1 in the treated animals (Figure 5A).

In order to validate Pter’s capability to activate the translocation of NRF2 to the nucleus to provide better antioxidant defence, we decided to perform in vitro assays using HREC cells. Incubation with non-cytotoxic Pter concentrations showed a protective effect from oxidative stress induced by high glucose exposure on HREC after 24 h (Figure 5B,C). Treatment with stilbene promoted maximum NRF2 translocation into the nucleus in HREC after 6 h of incubation (Figure 5D) and increased *NQO1* gene expression (Figure 5E).

The tight control on NRF2 translocation levels is subjected to two mechanisms mediated through Kelch-like ECH-associated protein 1 (KEAP1), which presents NRF2 to the Cullin3/RING box protein 1 complex for its degradation by proteasome 26S, and GSK3β that phosphorylates NRF2 prior to E3 ligase adapter β-TrCP and Cullin1/RING box recruitment for ubiquitin-proteasome degradation [60,61]. In vitro [62,63] and in vivo [64] experiments have shown Pter’s capacity to activate NRF2 through KEAP1 release. Nonetheless, whether Pter is able to modulate NRF2 through GSK3β remains unknown.

GSK3α and GSK3β are in an inactive state due to AKT-mediated phosphorylation. AKT phosphorylates a myriad of protein substrates, including E2 ubiquitin ligases, transcription factors, protein and lipid kinases, metabolic enzymes, etc., which shows that the main role of AKT is not just to regulate one physiological process, but to control multiple targets and functions. In fact, inhibition of GSK3 by AKT-phosphorylation is a clue to the regulation of transcription factors such as NRF2 [65], an essential actor for DR development. Many studies have reported that AKT is involved in the PI3K antioxidant signalling pathway and that molecular pathway PI3K/AKT/GSK3β/NRF2 activation is a mighty protector against oxidative damage [19,20,21,65]. Consistently with these reports, we observed that Pter increased the expression of *NQO1* in HREC, a downstream event of NRF2 activation, which was reversed by inhibitors of PI3K and GSK3β, BEZ235 and lithium chloride, respectively (Figure 5E). Additionally, in the present study activation of the PI3K/AKT/GSK3β/NRF2 pathway was detected with Pter treatment in diabetic rabbit retinas (Figure 5A–C). Hence our findings indicate that Pter is an NRF2 activator through PI3K/AKT/GSK3β pathway modulation, and is capable of preventing the early molecular changes associated with DR development.

## 5. Conclusions

Our study demonstrates for the first time using in vivo studies that early neuro-retinal damage caused by hyperglycaemia can be prevented by employing non-toxic and bioavailable concentrations of Pter. The PI3K/AKT/GSK3β/NRF2 molecular pathway plays a critical role in Pter protection against ROS, such as H_2_O_2_ produced in diabetic retinas. PI3K activation induces GSK3β inhibition via AKT, which activates NRF2 nucleus translocation, the expression of antioxidant enzymes and redox homeostasis regulation by counteracting the oxidative stress induced by hyperglycaemia in the retina. Based on the results of this report, we propose Pter to be a potential nutraceutical for lowering the risk of DR development. 

## Figures and Tables

**Table 1:** In vivo toxicity of pterostilbene (50 mg/kg).

	Control	Diabetic	Treated
ALT (U/L)	49.0 ± 3.6	70.8 ± 6.3 *	50.3 ± 2.6 †
GOT	16.5 ± 0.9	20.7 ± 2.7	22.7 ± 1.2
Alkaline phosphatase (U/L)	102.0 ± 8.9	137.7 ± 6.1 *	53.0 ± 4.9 ** †††
Total bilirubin (mg/dL)	0.1 ± 0.01	0.1 ± 0.02	0.2 ± 0.02
Albumin (g/L)	47.0 ± 0.7	44.2 ± 1.0	35.3 ± 0.9 *** †††
Total protein (g/L)	60.8 ± 2.2	55.2 ± 1.1	67.3 ± 1.9
Chloride (mmol/L)	102.3 ± 2.3	96.6 ± 1.2	100.7 ± 2.7
Blood Urea Nitrogen (mg/dL)	22.7 ± 2.5	20.9 ± 1.7	20.1 ± 2.8
Creatinine (mg/dL)	0.9 ± 0.1	1.0 ± 0.1	0.9 ± 0.1
Calcium (mg/dL)	11.9 ± 0.1	11.9 ± 0.3	12.1 ± 0.3
Phosphorus (mg/dL)	5.5 ± 0.2	6.1 ± 0.2	5.9 ± 0.4
Sodium (mmol/L)	140.8 ± 0.5	138.4 ± 0.4	138.3 ± 0.3
Potassium (mmol/L)	3.7 ± 0.1	4.1 ± 0.1	3.9 ± 0.1
HDL cholesterol (mg/dL)	19.2 ± 2.8	19.0 ± 2.3	25.4 ± 3.8
Total cholesterol (mg/dL)	41.5 ± 4.4	42.4 ± 3.9	63.7 ± 14.2
Urea (mg/dL)	48.5 ± 5.4	44.8 ± 3.6	43.0 ± 6.1
Uric acid (mg/dL)	3.6 ± 0.4	4.2 ± 0.3	4.4 ± 0.3

Biochemical measurements were taken with plasma samples six weeks after diabetes induction. Data are presented as mean ± SD (at least *n =* 4 in each group). Differences among groups were assessed by a one-way ANOVA, followed by Tukey’s test. * *p* < 0.05; ** *p* < 0.01; *** *p* < 0.001 versus the control group. † *p* < 0.05; ††† *p* < 0.001 versus the diabetic group.
